# Laparoscopic Partial Hepatectomy: Animal Experiments

**DOI:** 10.1155/DTE.2.39

**Published:** 1995

**Authors:** Haruhiro Inoue, Narihide Goseki, Yousuke Izumi, Kimiya Takeshita, Hiroshi Nakamura, Mitsuo Endo

**Affiliations:** First Department of Surgery Tokyo Medical and Dental University 1-5-45 Yushima, Bunkyo-ku Tokyo 113 Japan

## Abstract

As a first step in firmly establishing laparoscopic hepatectomy, we introduce a porcine model of laparoscopic partial hepatectomy. This procedure has been successfully performed under the normal-pressure or low-pressure pneumoperitoneum condition supported by the full-thickness abdominal wall lifting technique. An ultrasonic dissector combined with electrocautery, newly developed by Olympus Optical Corporation (Japan) was effectively utilized in facilitating safe and smooth incisions into the liver parenchyma. Although indications for this procedure seem to be limited only to peripheral lesions and not to central lesions, clinical application of this method may be useful for some patients in the near future.


*Diagnostic and Therapeutic Endoscopy*, 1995, Vol. 2, pp. 39–42
Reprints available directly from the publisher
Photocopying permitted by license only

© 1995 Harwood Academic Publishers GmbH
Printed in Singapore

### Laparoscopic Partial Hepatectomy: Animal Experiments[1]

HARUHIRO INOUE, NARIHIDE GOSEKI, YOUSUKE IZUMI, KIMIYA TAKESHITA, HIROSHI NAKAMURA and MITSUO ENDO

*First Department of Surgery, Tokyo Medical and Dental University, 1-5-45 Yushima, Bunkyo-ku, Tokyo 113, Japan*

*(Received December 13, 1993; revised March 6, 1995)*

As a first step in firmly establishing laparoscopic hepatectomy, we introduce a porcine model of laparoscopic partial hepatectomy. This procedure has been successfully performed under the normal-pressure or low-pressure pneumoperitoneum condition supported by the full-thickness abdominal wall lifting technique. An ultrasonic dissector combined with electrocautery, newly developed by Olympus Optical Corporation (Japan) was effectively utilized in facilitating safe and smooth incisions into the liver parenchyma. Although indications for this procedure seem to be limited only to peripheral lesions and not to central lesions, clinical application of this method may be useful for some patients in the near future.

**KEY WORDS:** laparoscopic hepatectomy, laparoscopic partial hepatectomy, ultrasonic dissector, low-pressure pneumoperitoneum

### INTRODUCTION

Hepatocellular carcinoma or metastatic lesions of the liver present near the surface of the liver in some cases. Such small lesions, if single or localized, exhibits the potential for curable resection by partial hepatectomy. This surgical procedure is not particularly complicated, since it is not accompanied by any reconstructive process. Thus it seems to be a relatively good candidate for minimal-access surgery. In an effort to clarify the possibility of clinical application of laparoscopic partial hepatectomy, therefore, we undertook an experimental study using a porcine model.

### MATERIALS AND METHODS

### Laparoscopic partial hepatectomy

A high-pressure pneumoperitoneum of 12–15 mmHg is primarily achieved under general anesthesia through an endotracheal-intubation tube. Three 10- to 12-mm O.D. working trocars are inserted near the umbilicus and on each side of abdomen at the level of umbilicus. Two additional 5-mm O.D. trocars are also anchored in both subcostal areas for retracting the liver lobes (1). An abdominal wall retractor is inserted around the umbilicus and into the abdominal cavity to just above the target liver lobe under direct laparoscopic visual control. This abdominal wall retraction creates normal- or low-intraabdominal pressure, which maintains a good visual field inside the abdominal cavity. This is comparable to the visualization possible with a high-pressure pneumoperitoneum (2). In order to delineate the resected line clearly, the liver surface including the lesion is marked by coagulation using the tip of the electrocoagulator. The distal part of the resected liver lobe is grasped and retracted by Endo-Babcock forceps. The liver parenchyma is dissected using the ultrasonic dissector combined with electrocautery (Prototype, Ultrasonic Surgical Unit, Olympus) (Fig. 1 a, b) (3). Exposed small vessels are coagulated and divided with this ultrasonic dissector. The effectiveness can be assessed by comparing it with the conventional bipolar-coagulation forceps. Larger vessels are clipped with an Endoclip (USSC) and then cut with scissors. The cut edges of the vessels are reinforced by a clipping device that facilitates complete hemostasis. By repeating this procedure we can accomplish partial hepatectomy. In total, twenty one pigs received this laparoscopic procedure (Fig. 2).

---

Address for correspondence: Haruhiro Inoue, M.D., First Department of Surgery, Tokyo Medical and Dental University, 1-5-45 Yushima, Bunkyo-ku, Tokyo 113, Japan.

[1]The technical feasibility of laparoscopic partial hepatectomy was evaluated by animal experiments. Partial hepatectomy of the distal part of all lobes except for segments 1 and 7 was successfully performed under low-pressure pneumoperitoneum.

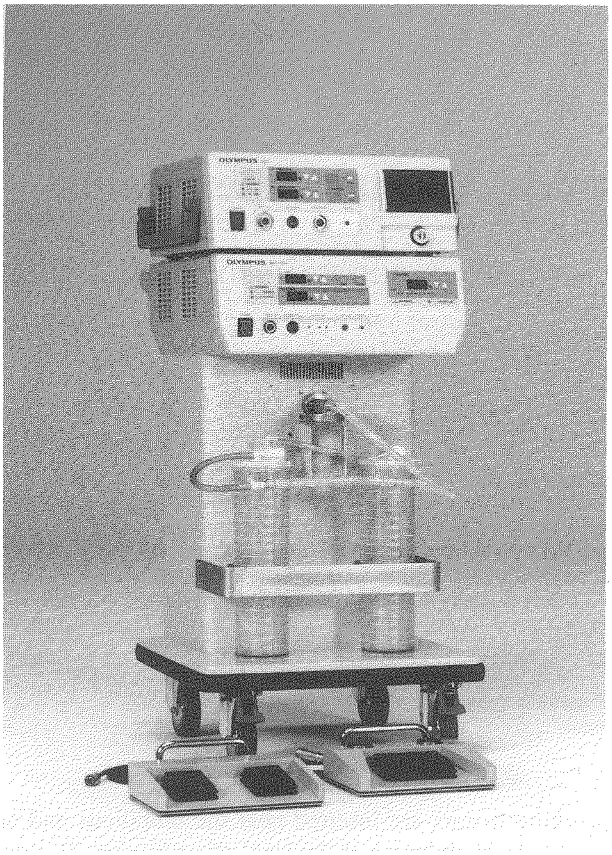

a

### RESULTS

By employing the total abdominal wall lifting technique combined with a 0–4-mmHg pneumoperitoneum, a good laparoscopic visual field sufficient to gain access to the anterior surface of the liver lobes could be created in all twenty one pigs. Distal-half resection of the liver lobes was successfully carried out in all segments except for segments 1 and 7. These latter segments were anatomically located in the dorsal part of the liver and could not readily be approached laparoscopically. During the dissection of the liver parenchyma, the ultrasonic dissector was more effectively utilized to control bleeding from the cut edge of the liver parenchyma than the bipolar-coagulation forceps (Fig. 3a). After skeletonizing the Glisson sheathes on the cutting plane of the liver using the ultrasonic dissector, the exposed small vessels were coagulated. They were cut simultaneously by electrocautery generating at the tip of ultrasonic dissector. The larger vessels and bile ducts were ligated by Endoclip and then cut by Endoscissors (Fig. 3b). We encountered no uncontrollable bleeding and no visible injury to the main bile duct during this procedure (Fig. 3c). Each resected specimen was easily extracted from the abdominal cavity using an Endopouch (Fig. 3d).

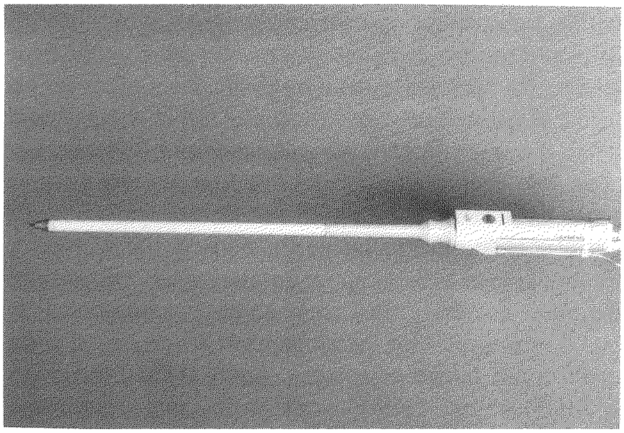

b

**Figure 1a** Entire view of ultrasonic surgical unit (Prototype, Olympus Optical Corporation). **b** Tip of the ultrasonic dissector, which simultaneously serves as an ultrasonic dissector and as an electrocautery.

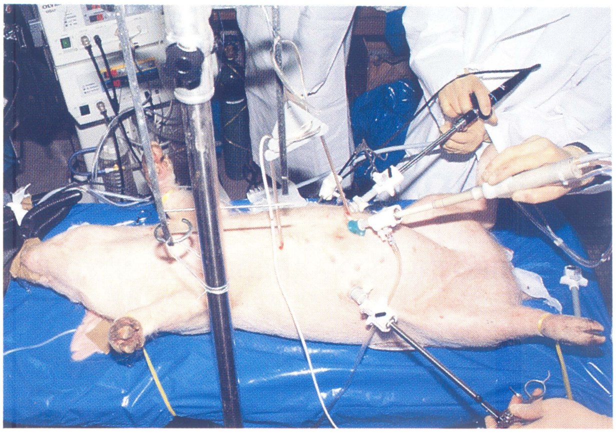

**Figure 2** Laparoscopic partial hepatectomy under way.

Time spent on performing this laparoscopic procedure is about two times longer than time of partial resection during open surgery.

### DISCUSSION

As is evident from the vast clinical experience obtained from laparoscopic cholecystectomy, minimal access surgery has gained considerable popularity. We set out to enlarge the application of these laparoscopic techniques to include simple hepatic resection. Some small hepatomas or metastatic diseases of the liver located in the superficial part maybe suitable for partial hepatectomy. The surgical procedure for partial resection is less complicated because it involves no reconstruction. It appears to be an amenable candidate to a laparoscopic approach.

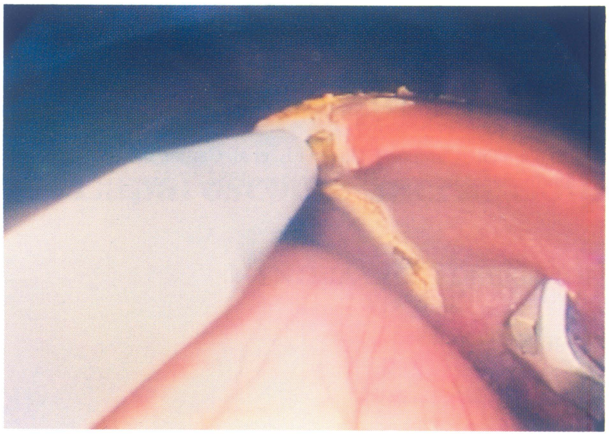

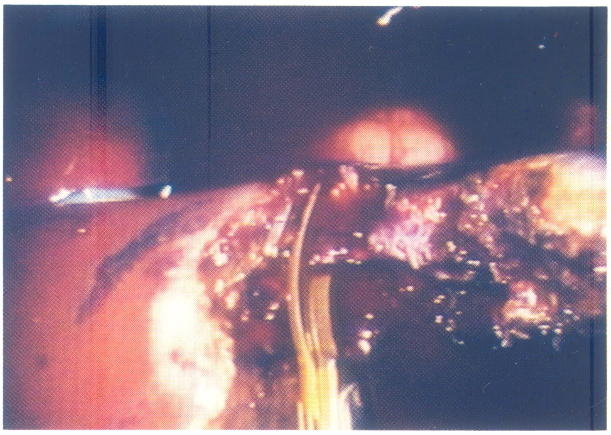

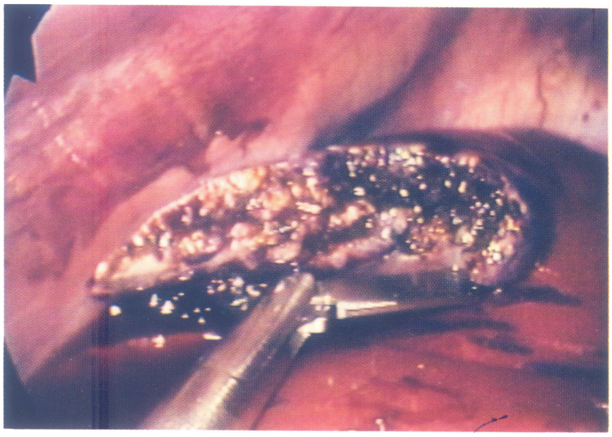

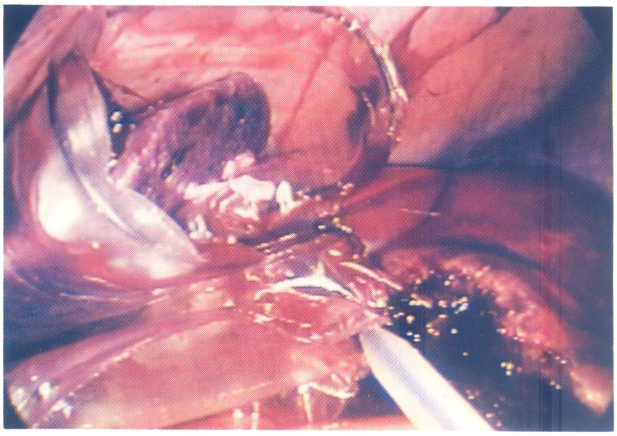

a b c d

**Figure 3** Laparoscopic view of partial hepatectomy of segment 8. **a** Liver parenchyma is dissected by aspiration and coagulation of the ultrasonic dissector. **b** Exposed vessels and bile ducts are clipped and divided. **c** Cut edge of the liver. Complete hemostasis was achieved. **d** Resected specimen is extractyed by Endopouch.

When a liver parenchyma resection is attempted, using a technique other than high-pressure pneumoperitoneum it is especially important to eliminate the risk of carbon dioxide embolism migrating from the cut edge of the liver parenchyma (4,5). Accordingly, we instituted the abdominal wall lifting method combined with null- or low-pressure pneumoperitoneum which has been advocated by some Japanese surgeons including us (2).

Concerning the dissection of liver parenchyma, the ultrasonic dissector (Olympus, Japan) was effective in controlling bleeding from the cut edge. This apparatus worked simultaneously as either an ultrasonic aspirator or as an electrocoagulator simply by controlling the switch on the hand piece of the dissector. In this experimental study, we thus confirmed that the ultrasonic dissector is superior to bipolar forceps which mechanically crushed the liver tissue but could not coagulate the tissue vasculature sufficiently.

Our animal experience suggested that both abdominal wall lifting and utilization of the ultrasonic dissector were the key factors for safe and successful partial hepatectomy. We would like to emphasize that this technique should be applied only to peripheral lesions and not to central lesions.

From this experimental study, we conclude that if the appropriate candidate for this procedure were carefully selected prior to clinical application, partial hepatectomy may be possible in clinical cases.

### REFERENCES

1. Inoue H, Muraoka Y, Takeshita K, et al. A balloon-tipped liver lobe retractor. Surg Endosc, 1993;6:561.
2. Inoue H, Muraoka Y, Takeshita K, et al. Low-pressure pneumoperitoneum for laparoscopic surgery using a disposable, flexible abdominal wall retractor. Digest Endosc, 1993;5:289–292
3. Yamakawa T, Kano N, Sakai S, et al. Preliminary experience using an ultrasonic aspirator for laparoscopic cholecystectomy. Endoscopy, 1992;24:721–723
4. Root B, Levy MN, Pollack S, et al. Gas embolism death after laparoscopy delayed by "trapping" in portal circulation. Anesth Analg, 1978;57:232–237
5. Yacoub OF, Cardona I, Coveler L, et al. Carbon dioxide embolism during laparoscopy. Anesthesiology, 1982;57:533–535

